# Association between urinary albumin‐to‐creatinine ratio within normal range and hypertension among adults in the United States: Data from the NHANES 2009–2018

**DOI:** 10.1002/clc.24012

**Published:** 2023-04-05

**Authors:** Li Ming, Duan Wang, Yong Zhu

**Affiliations:** ^1^ Department of Pediatrics, Xinqiao Hospital Army Medical University Chongqing China; ^2^ Department of Rehabilitation Children's Hospital of Chongqing Medical University Chongqing China; ^3^ Department of Pediatric Center University‐Town Hospital of Chongqing Medical University Chongqing China

**Keywords:** albuminuria, hypertension, NHANES, urinary albumin‐to‐creatinine ratio

## Abstract

**Background:**

Recently it was suggested that urine albumin‐to‐creatinine ratio (uACR), even within the normal range, can be associated with hypertension, but only a few studies have examined. Therefore, this study aimed to determine the association between normal range uACR and the prevalence of hypertension.

**Methods:**

The research used data from the 2009 to 2018 National Health and Nutrition Examination Survey, which included 14,919 participants. We defined the uACR as the amount of albumin (mg/dL) divided by creatinine (g/dL) in randomly voided urine. Hypertension was defined as mean systolic blood pressure ≥130 mmHg, or diastolic ≥80 mmHg, or were taking hypertension medication or were informed of a hypertension diagnosis by a physician/health professional.

**Results:**

In multivariable‐adjusted models, per 5 mg/g uACR increment, the hypertension prevalence increased 1.31‐fold (OR, 1.31; 95% CI 1.23–1.40), the odds [95% confidence interval (CI)] for hypertension prevalence were 2.25 (1.86–2.72) for those in the highest quartile compared to those in the lowest quartile. The nonlinear relationship between hypertension prevalence and uACR was found by visually assessing images (*p* for nonlinearity<.001). In addition, in the subgroup analysis stratified by body mass index, the lower the BMI, the stronger the association between uACR and hypertension prevalence.

**Conclusions:**

Even within the normal range, subtly elevated uACR was associated with an increased prevalence of hypertension in the USA general population, and this association may be enhanced in individuals with low BMI. Further research is needed to assess the clinical applicability of these findings.

## Graphical abstract

This study assessed the association between uACR levels and the prevalence of hypertension in adults in the United States, using data from NHANES 2009–2018. To conclude that even within the normal range, subtly elevated uACR was associated with an increased prevalence of hypertension in the USA general population, and this association may be enhanced in individuals with low BMI.

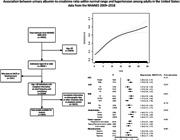

## 1 BACKGROUND

Chronic kidney disease (CKD) is a significant public health issue,^1^ About 37 million adult Americans have CKD, according to the Centers for Disease Control and Prevention and prevalence projections indicate that they will rise as the population ages and the obesity and diabetes epidemics spread,^2^, ^3^ although end‐stage renal disease (ESKD) development is a serious outcome of CKD, cardiovascular problems are mostly responsible for the high morbidity and mortality in these people.^4^, ^5^

Individualized hypertension control targets are an important intervention for CKD management in primary care.^6^, ^7^ In addition to controlling the risk factors currently known to be associated with blood pressure, we also need to focus on the role of other biomarkers in blood pressure. Albuminuria was closely associated with the risks of cardiovascular diseases (CVD) and several chronic diseases,^8^, ^9^, ^10^ and changes in albuminuria observed in trials of cardiorenal preventive therapies strongly correlate with clinical endpoints.^11^

The urinary albumin‐to‐creatinine ratio (uACR), which is a well‐known indicator of glomerular injury and a crucial diagnostic indicator of chronic kidney disease (CKD).^4^ UACR is a reliable method for monitoring the excretion of urine protein and has become a clinical qualitative and quantitative diagnostic index for proteinuria that can replace the traditional 24‐h urinary protein quantification.^12^ Given the negative effects of albuminuria on CVD and the ambiguity of intervention when individual urinary protein levels rise slightly in the normal range (<30 mg/g), it is important to investigate the relationship between uACR and hypertension. Furthermore, fewer studies have examined the associations between uACR and hypertension, and no relevant research has been conducted in the NHANES population.

Therefore, we investigated the association between uACR and hypertension in US adults using data from the 2009 to 2018 National Health and Nutrition Examination Survey (NHANES). We also looked at whether the association varies by participant characteristics like age, gender, BMI, behavioral risk factor, and comorbidities, which could help with future clinical management of uACR.

## 2 METHODS

### 2.1 Study design and population

The Centers for Disease Control and Prevention (CDC) of America conducts the NHANES on a 2‐year cycle, participants are noninstitutionalized individuals from the United States who are chosen through a complex stratified, multistage sampling design. In the current study, we used data from five NHANES cycles (2009–2010, 2011–2012, 2013–2014, 2015–2016, and 2017–2018) to investigate the association between uACR and blood pressure among NHANES participants. All information was obtained from the Public Data General Release file documents. The National Center for Health Statistics Ethics Review Board approved the NHANES, and all participants provided written informed consent before completing the NHANES.^13^

**Figure 1:**
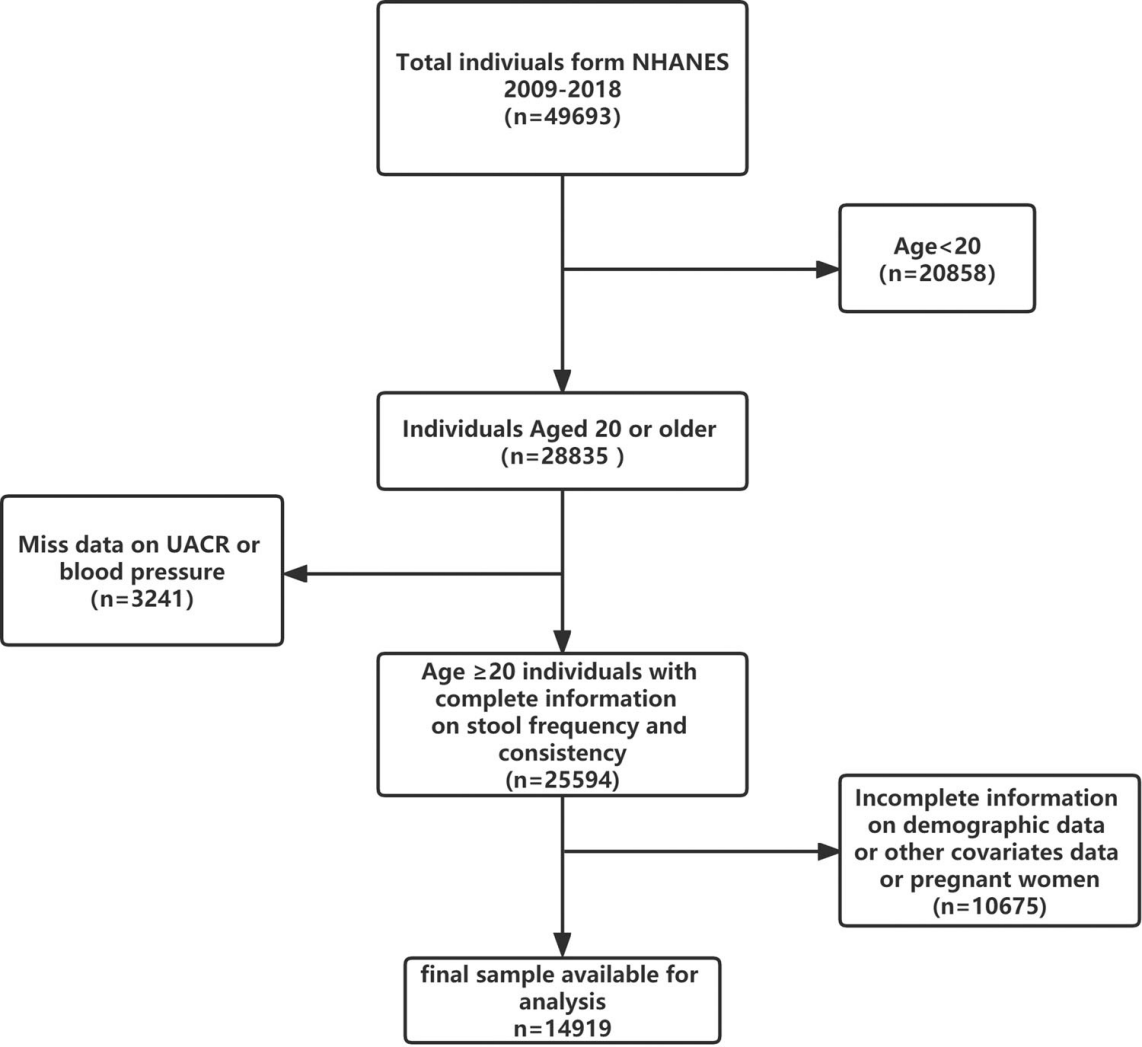
Flowchart of the sample selection from National Health and Nutrition Examination Survey 2009–2018.

In the present study, we analyzed NHANES participants (age ≥20 years) from five cycles of NHANES (2009–­2018), data on demographics, exams, lab tests, and questionnaires were gathered. Participants who have incomplete data on laboratory urine data, blood pressure, and pregnant women were excluded, participants had missing demographic data and other covariates data also excluded. After exclusions, this study contained a total of 14 919 participants aged ≥20 years with normal uACR (7605 female and 7314 male) (Figure 1).

### 2.2 Urinary albumin‐to‐creatinine ratio

Trained researchers collected 5 mL of spotted urine from each participant and sent frozen urine samples (−20°C) to the laboratory. The sample's stability was demonstrated at 5°C and temperatures less than or equal to −20°C.^14^ The solid‐phase fluorescent immunoassay was used to test urine albumin, while the enzymatic technique was used to quantify urine creatinine, in accordance with the NHANES's recommendations, the gold standard method was used to standardize and calibrate the amounts of urine albumin and creatinine.^15^, ^16^ uACR was calculated and reported in milligrams per gram (uACR = urine albumin/urine creatinine).

### 2.3 Outcome ascertainment

The main outcome variable, blood pressure (BP), was measured with a mercury sphygmomanometer by trained personnel following standardized protocols,^17^ following a 5‐min quiet rest in the seated position, three BP readings were taken consecutively, and the participant's maximum inflation level was determined. If the blood pressure measurement is interrupted or incomplete, a fourth measurement can be taken.

All systolic and diastolic blood pressure measurements were taken at the Mobile Examination Center. In the present study, we calculated the average of systolic and diastolic BP readings for further analyses, if they had only one BP reading, then, it was the final record. Hypertension was defined as a mean SBP ≥130 mmHg, or a mean DBP ≥80 mmHg, or were taking hypertension medication or were informed of a hypertension diagnosis by a physician/health professional.

### 2.4 Potential covariates

Demographic characteristics included age (20–39 years, 40–59 years, and ≥60 years), gender (female, male), race/ethnicity (non‐Hispanic white, non‐Hispanic black, Mexican American, other Hispanic, or others), levels of education (below high school, high school and above high school), poverty income ratio (<1.35, 1.35–1.85, >1.85). Dietary information on total sodium and total energy intake was collected by trained interviewers. Body mass index (BMI) categorized as under/normal weight (<25 kg/m^2^), over‐weight (25–29.9 kg/m^2^), and obese (≥30 kg/m^2^). Laboratory tests include Uric Acid (UA, mg/dL), total cholesterol (TC, mg/dL), and estimated glomerular filtration rate (eGFR, mL/min/1.73 m^2^). Assessments of behavioral risk factors included smoking, drinking, and physical exercise. Smoke exposure is measured by the level of serum cotinine (<0.05 ng/mL: unexposed or nonsmoker; 0.05–10 ng/mL: exposed but not an active smoker; >10 ng/mL: active smoker).^18^, ^19^ The drinking status was divided into three categories: never (less than 12 drinks in a year), former (more than 12 drinks in a year but no longer drinking), and current (more than 12 drinks in a year but still drinking).^20^ Physical activity status was classified as vigorous, moderate and inactive.^21^ Diabetes was defined as being informed by doctor/health professional about the diagnosis of diabetes and/or a glycosylated hemoglobin measurement of ≥6.5%.^22^

### 2.5 Statistical analysis

The participants were characterized using descriptive statistics, where continuous variables were expressed as mean standard deviation and categorical variables as frequency or as a percentage. The one‐way ANOVA (normal distribution) and Kruscal–Wallis H (skewed distribution) tests were used to compare continuous variables between the various groups. The *χ* ^2^ test or Fisher's exact test was used to compare categorical variables between the various groups. Multivariate logistic regression analysis was used to evaluate blood pressure and hypertension based on the uACR (continuous and categorical variables). The covariate adjustment was determined by the following principle: when covariates were added to this model, the matched odds ratio changed by at least 10%. P for trend tests were performed by rerunning the corresponding regression models with the quartiles of uACR as a continuous variable. In addition, to characterize the shape of the relationship between uACR and the main outcome, a generalized additive model and fitted smoothing curve were used. The log‐likelihood ratio test was used to determine whether a threshold exists by comparing a one‐linev (nonsegmented) model to a segmented regression model. The inflection point connecting the segments was determined using two‐step recursive methods based on the model with the highest likelihood. Subgroup analyses were carried out using stratified linear regression models to detect effect modification.

According to recommendations from the National Center for Health Statistics, appropriate sampling weights (i.e., 2‐year mobile examination sample weights 0.2) were created to take into consideration the complicated survey design and produce nationally representative estimates.^23^ All of the analyses were performed with the statistical software packages R (http://www.R-project.org, The R Foundation). *p* < .05 (two‐sided) were considered statistically significant.

## 3 RESULTS

We included 14 919 participants in our analysis. The average age of the participants was 49.7 ± 17.6 years, with a male versus female ratio (49.02% vs. 50.65%). The median uACR of the participants was 6.3 mg/g (interquartile range, 4.3–10.0 mg/g). The weighted prevalence of hypertension was 35.8%. The baseline characteristics of participants according to uACR level from the NHANES (2009–2018) are shown in Table 1. Participants with a higher uACR were generally female, older, and had lower annual household income, education, physical activity and higher BMI, compared to those with lower uACR. For dietary intake, the individuals with lower uACR consumed more calories and sodium, and have more physical activity, in addition, higher uACR levels were associated with higher rates of diabetes and hypertension, in crude analyses, the incidence of hypertension, systolic and diastolic blood pressure varied significantly across quartiles of uACR (*p* < .001).

**Table 1:** Characteristics of participants by quartiles of uACR in the 2009–2018 continuous NHANES.

Characteristics	uACR quartiles				*p* Value
	Quartile 1	Quartile 2	Quartile 3	Quartile 4	
No of participants	3726	3732	3664	3797	
Age, year					<.001
20–39	1690 (45.4%)	1402 (37.6%)	1168 (31.9%)	936 (24.7%)	
40–59	1352 (36.3%)	1363 (36.5%)	1260 (34.4%)	1244 (32.8%)	
>=60	684 (18.4%)	967 (25.9%)	1236 (33.7%)	1617 (42.6%)	
Sex					<.001
Female	1207 (32.4%)	1905 (51.0%)	2162 (59.0%)	2331 (61.4%)	
Male	2519 (67.6%)	1827 (49.0%)	1502 (41.0%)	1466 (38.6%)	
Race					<.001
Mexican American	428 (11.5%)	524 (14.0%)	541 (14.8%)	553 (14.6%)	
Non‐Hispanic Black	889 (23.9%)	725 (19.4%)	615 (16.8%)	732 (19.3%)	
Non‐Hispanic White	1620 (43.5%)	1660 (44.5%)	1654 (45.1%)	1683 (44.3%)	
Other Hispanic	319 (8.6%)	375 (10.0%)	382 (10.4%)	402 (10.6%)	
Other Race—Including multi‐Racial	470 (12.6%)	448 (12.0%)	472 (12.9%)	427 (11.2%)	
Educational level					<.001
Below high school	615 (16.5%)	645 (17.3%)	741 (20.2%)	831 (21.9%)	
High school	794 (21.3%)	793 (21.2%)	800 (21.8%)	899 (23.7%)	
Above high school	2317 (62.2%)	2294 (61.5%)	2123 (57.9%)	2067 (54.4%)	
PIR					<.001
<1.35	1079 (29.0%)	1126 (30.2%)	1174 (32.0%)	1245 (32.8%)	
1.35–1.85	414 (11.1%)	449 (12.0%)	424 (11.6%)	489 (12.9%)	
>1.85	2233 (59.9%)	2157 (57.8%)	2066 (56.4%)	2063 (54.3%)	
BMI, kg/m^2^					<.001
Under/normal weight	961 (25.8%)	1063 (28.5%)	1056 (28.8%)	1081 (28.5%)	
Overweight	1354 (36.3%)	1276 (34.2%)	1104 (30.1%)	1155 (30.4%)	
Obese	1411 (37.9%)	1393 (37.3%)	1504 (41.0%)	1561 (41.1%)	
Energy, Kcal	2254.9 ± 848.0	2082.5 ± 833.0	2002.2 ± 802.0	1944.9 ± 769.3	<.001
Sodium, mg	3779.6 ± 1547.9	3473.4 ± 1518.1	3344.0 ± 1452.8	3236.7 ± 1380.2	<.001
UA, mg/dL	5.7 ± 1.3	5.4 ± 1.3	5.3 ± 1.4	5.3 ± 1.5	<.001
TC, mg/dL	190.1 ± 39.2	193.7 ± 41.5	192.8 ± 40.0	192.1 ± 41.7	.001
eGFR	95.2 ± 20.3	97.3 ± 20.8	95.7 ± 21.7	92.7 ± 23.8	<.001
Alcohol intake					<.001
Never	373 (10.0%)	444 (11.9%)	519 (14.2%)	570 (15.0%)	
Former	450 (12.1%)	501 (13.4%)	499 (13.6%)	587 (15.5%)	
Current	2903 (77.9%)	2787 (74.7%)	2646 (72.2%)	2640 (69.5%)	
Smoke exposure					.036
Unexposed or nonsmoker	1999 (53.7%)	2133 (57.2%)	2074 (56.6%)	2144 (56.5%)	
Exposed but not an active smoker	789 (21.2%)	752 (20.2%)	751 (20.5%)	797 (21.0%)	
Active smoker	938 (25.2%)	847 (22.7%)	839 (22.9%)	856 (22.5%)	
Physical activity					<.001
Inactive	1554 (41.7%)	1698 (45.5%)	1777 (48.5%)	2060 (54.3%)	
Moderate	949 (25.5%)	1020 (27.3%)	1073 (29.3%)	1073 (28.3%)	
Vigorous	1223 (32.8%)	1014 (27.2%)	814 (22.2%)	664 (17.5%)	
Lipoprotein‐lowering drugs					<.001
No	3241 (87.0%)	3110 (83.3%)	2904 (79.3%)	2773 (73.0%)	
Yes	485 (13.0%)	622 (16.7%)	760 (20.7%)	1024 (27.0%)	
Antihypertensive drugs					<.001
No	3554 (95.4%)	3533 (94.7%)	3394 (92.6%)	3522 (92.8%)	
Yes	172 (4.6%)	199 (5.3%)	270 (7.4%)	275 (7.2%)	
Hypertension					<.001
No	2662 (71.4%)	2461 (65.9%)	2113 (57.7%)	1775 (46.7%)	
Yes	1064 (28.6%)	1271 (34.1%)	1551 (42.3%)	2022 (53.3%)	
DM					<.001
No	3472 (93.2%)	3354 (89.9%)	3161 (86.3%)	3012 (79.3%)	
Yes	254 (6.8%)	378 (10.1%)	503 (13.7%)	785 (20.7%)	
SBP, mmHg	70.0 ± 10.3	70.5 ± 10.5	71.0 ± 11.4	71.2 ± 12.3	<.001
DP, mmHg	118.6 ± 13.4	120.3 ± 15.0	123.0 ± 16.9	127.6 ± 19.5	<.001

*Note*: Quartile 1: 4.3 mg/g; Quartile 2: 4.3–6.3 mg/g; Quartile 3: 6.3–10 mg/g; Quartile 4: 10 mg/g. Data are presented as number (%) or mean ± standard deviation.

Abbreviations: BMI, body mass index; DM, diabetes mellitus; DP, diastolic blood pressure; eGFR, estimated glomerular filtration rate; PIR, poverty income ratio; SBP, systolic blood pressure; TC, total cholesterol; UA, uric acid; uACR, urinary albumin‐to‐creatinine ratio.

Table 2 multivariate analyses show the relationship between uACR and the main outcomes in the included participants. After adjustment for age, sex, race, education, BMI, Energy, Sodium, UA, TC, and eGFR, there was a significant positive correlation between uACR and both systolic and diastolic blood pressures and prevalence of hypertension, similar results were found after further adjustment for all the covariates as defined above. For per 5 mg/g uACR increment, systolic blood pressure (*β*: 2.14; 95% CI 1.78–2.47) increased by 2.14 mmHg, diastolic blood pressure (*β*: 0.71; 95% CI 0.50–0.92) increased by 0.73 mmHg, and the hypertension prevalence increased 1.31‐fold (OR, 1.31; 95% CI 1.23–1.40). When continuous uACR was converted to quartiles, within the normal uACR range, the highest uACR quartile group had a 6.46 mmHg increase in systolic blood pressure (*β*: 6.46; 95% CI 5.55–7.36), a 2.63 mmHg increase in diastolic blood pressure (*β*: 2.63; 95% CI 1.94–2.32) and the risk of hypertension (OR, 2.25; 95% CI 1.86–2.72). increased 2.25‐fold compared to the lowest quartile group. There was a trend for higher *β* of blood pressure and OR of hypertension among participants in the higher quartile of uACR relative to the lower quartile (*p* for trend <.001).

**Table 2:** Weighted *β*/OR (95% confidence intervals) for relationship between uACR and blood pressure/hypertension in different models among US adult.

uACR, mg/g	Blood pressure β (95% CI)/Hypertension OR (95% CI), *p* Value		
	Model Ⅰ	Model Ⅱ	Model Ⅲ
SBP, mmHg			
Continuous (per 5 mg/g)	2.63(2.26–2.97)<.001	2.14(1.81–2.48)<.001	2.14(1.78–2.47)<.001
Q1	Reference	Reference	Reference
Q2	1.71(0.78–2.65)<.001	2.01(1.17–2.85)<.001	2.01(1.17–2.85)<.001
Q3	4.19(3.31–5.07)<.001	3.99 ((3.38–4.79)<.001	3.66(3.17–4.80)<.001
Q4	7.45(6.49–8.41)<.001	6.48(5.56–7.37)<.001	6.46(5.55–7.36)<.001
*p* for trend	<.001	<.001	<.001
DP, mmHg			
Continuous (per 5 mg/g)	0.22(0.01–0.43)<.001	0.63(0.43–0.84)<.001	0.71(0.50–0.92<.001
Q1	Reference	Reference	Reference
Q2	0.33(−0.39–1.04).373	1.08(0.42–1.73).002	1.18(0.53–1.84)<.001
Q3	1.08(0.43–1.74).002	2.30(1.66–2.94)<.001	2.46(1.82–3.09)<.001
Q4	0.75(0.32–1.48).044	2.37(1.69–3.06)<.001	2.63(1.94–3.32)<.001
*p* for trend	.007	<.001	<.001
Hypertension			
Continuous (per 5 mg/g)	1.39(1.32–1.47)<.001	1.35(1.26–1.44)<.001	1.31(1.23–1.40)<.001
Q1	Reference	Reference	Reference
Q2	1.28(1.11–1.48).001	1.38(1.17–1.62)<.001	1.31(1.11–1.56)<.001
Q3	1.71(1.48–1.98)<.001	1.68(1.41–2.00)<.001	1.56(1.32–1.86)<.001
Q4	2.61(2.24–3.05)<.001	2.46(2.04–2.97)<.001	2.25(1.86–2.72)<.001
*p* for trend	<.001	<.001	<.001

*Note*: Values are weighted *β*/OR (95% CIs) unless otherwise indicated. uACR, albumin‐to‐creatinine ratio. Model 1 was adjusted for none. Model 2 was adjusted for age, sex, race, education, BMI, Energy, Sodium, UA, TC, eGFR. Model 3 was adjusted for age, sex, race, BMI, Energy, Sodium, UA, TC, eGFR, DM, alcohol intake, smoke exposure, total physical activity, lipoprotein‐lowering drugs, antihypertensive drugs.

Abbreviations: DP, diastolic blood pressure; SBP, systolic blood pressure; uACR, urinary albumin‐to‐creatinine ratio.

**Figure 2:**
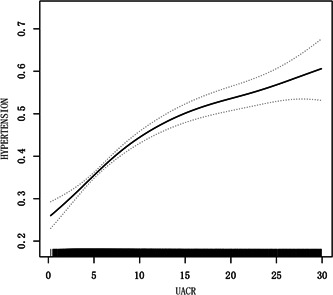
Association between urine albumin‐to‐creatinine ratio and the prevalence of hypertension. The solid line and dashed line represent the estimated values and their corresponding 95% confidence interval.

uACR and the prevalence of hypertension were positively correlated, according to the generalized additive model's results and fitted smoothing curve (Figure 2). The nonlinear relationship between hypertension prevalence and uACR was found by visually assessing images (*p* for nonlinearity<.001), further analysis found that the inflection point of the threshold effect was 7.65 mg/g of uACR, below the threshold, the prevalence of hypertension increased significantly for each 1 mg/g increase in uACR (OR, 1.14; 95% CI 1.11–1.17) and then slowly increased above the threshold (OR, 1.04; 95% CI 1.02–1.05).

**Figure 3:**
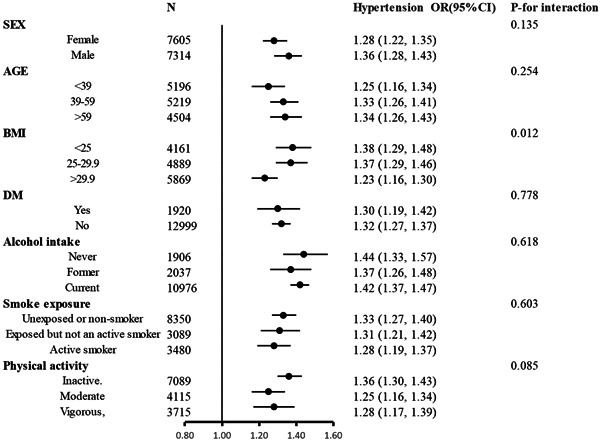
Stratified analyses by potential modifiers of the association between urine albumin‐to‐creatinine ratio and the prevalence of hypertension.

A stratified analysis of participants was performed to assess the relationship between uACR (continuous variable) and hypertension in various subgroups (Figure 3). An interesting finding was that the lower the BMI, the higher the OR of uACR to the incidence of hypertension (*p* for interactions = 0.012), other than BMI, age, gender, smoking status, drinking status, physical activity and diabetes did not significantly change the association between uACR and hypertension prevalence (*p* for all interactions >.05).

## 4 DISCUSSION

The present study utilized the population‐based cross‐sectional resources of NHANES to detect the correlation between uACR levels and prevalence of hypertension in adults, which, to our knowledge, was the first time to examine such a relationship in this population. We found that uACR within the normal range was positively correlated with systolic and diastolic blood pressures and the prevalence of hypertension even after multivariable adjustment. The nonlinear relationship between hypertension prevalence and uACR was found by visually assessing images. The optimal cutoff point of the association of uACR with hypertension was 7.65 mg/g, on each side of the optimal uACR value, the magnitude of the correlation between uACR and the prevalence of hypertension was different. Furthermore, the stratified analysis showed that the association between uACR levels with hypertension was much stronger among nonobese individuals compared with obese people.

Although the mechanisms underlying the association of uACR with hypertension are largely unknown, there are some possible explanations. Systemic and glomerular vascular endothelial dysfunction may be a potential physiological link between albuminuria and hypertension.^24^ Increased albuminuria indicates extended microvascular endothelial cellular injury,^25^ which could predispose to atherogenic lipoprotein accumulation in the subendothelial cell space.^26^ Thus, increased albuminuria might reflect glomerular and/or systemic vascular endothelial dysfunction that precedes the development of hypertension in humans. On the other hand, reduced nephron numbers have been implicated as a risk factor for developing hypertension.^27^ An autopsy study suggests that hypertensive individuals may in fact have fewer nephron numbers than normotensive individuals.^28^ In individuals who have fewer nephrons numbers, the intraglomerular pressure and glomerular filtration of the remaining glomeruli are raised to make up for the kidney's lower glomerular filtration rate, which causes albuminuria to rise.^29^ Elevated albuminuria might therefore in turn be a sign of reduced nephron numbers.

There has been growing evidence in recent years that urine albumin excretion, even when it is within the normal range, is linked to an increased risk of hypertension. The Nurse's Health Study^30^ and the Atherosclerosis Risk in Communities Study^31^ suggested that elevated uACR within the normal range could be an independent predictive factor of hypertension in the USA population. Studies performed in Chinese,^32^ Japanese,^33^ Korean^34^, ^35^, ^36^ populations and in Indo‐Asian people^37^ also demonstrated a significant association of uACR, even within the normal range, with hypertension after adjusting for potential confounding factors. Our findings consistent with those of earlier research. Takase et al.^38^ thought that given the similarities of the Kaplan–Meier curves in the first to third quartiles, there could be a urinary albumin threshold for the development of hypertension, however, they could not determine the finding. In our study, we discovered a nonlinear association between the prevalence of hypertension and uACR using a smoothing curve and showed a threshold effect. The association between hypertension prevalence and uACR was significantly attenuated when uACR exceeded 7.65 mg/g, although the association was still statistically significant (OR, 1.04; 95% CI 1.02–1.05). In addition, we found that participants with higher uACR levels were more predisposed to be female, older, non‐Hispanic white, with low incomes, poor education, obesity, and diabetes. However, these results were obtained only from univariate estimates, and further observational and experimental studies are needed to elucidate these results.

Furthermore, the stratification analyses of age, gender, smoking status, drinking status, physical activity, and diabetes were conducted, and no significant interaction was found. This positive association persisted across subgroups stratified by these factors, demonstrating the robustness of our findings and increasing our confidence in the reliability of the results. But notably, in our BMI‐stratified subgroup, nonobese individuals showed a stronger association between uACR levels and hypertension than obese individuals (*p* for interactions = .012). However, our data do not suggest that uACR can more accurately identify those at high risk of incident hypertension among nonobese individuals. Concerns have been raised that interindividual differences in muscular mass have a significant influence on the uACR value via urinary creatinine excretion, that the uACR assessment overestimates urine albumin excretion in people who have low muscle mass, and therefore may not be associated with vascular disease.^39^ BMI is the most commonly used metric for measuring obesity, but it lacks the ability to provide information on fat distribution and distinguish fat accumulation from muscle.^40^ Therefore, further studies are warranted to elucidate the complex interaction between obesity and urinary albumin as well as the possible mediating role of obesity in the urinary albumin excretion−hypertension association.

Our research has its advantages. First, the sample is representative, and the sample size is sufficient. Second, we modified for potential confounders to produce more reliable results. Dietary sodium, for example, influences both hypertension and the degree of albuminuria. Previous studies did not include information on individual sodium intake, but we included this potential covariate in our analyses to strengthen our findings. However, our study does have some limitations that should be discussed. First, this study is cross‐sectional research, therefore it could not demonstrate the causation but only the association. Second, uACR measurement was evaluated only from a single spot urine specimen but not in the 24 h urine collection. However, previous studies have shown that the result of a single spot urine specimen has a close association with total amounts of urinary albumin excretion in 24‐h urine, and because the test is more convenient and cost saving, in collecting large epidemiological specimens, the use of single spot urine samples for testing uACR has been suggested as a trustworthy alternative.^41^ Finally, the average blood pressure that is determined by taking readings continuously for a short period of time might not accurately reflect the true state of blood pressure. In addition, genetic and environmental factors can be confounding factors that may affect the association results, we cannot ignore the influence of these factors.

## 5 CONCLUSION

In conclusion, our results suggest that even within the normal range, subtly elevated uACR was associated with an increased prevalence of hypertension in the USA general population. For individuals with high uACR, closer monitoring of blood pressure is highly recommended. However, whether uACR is a good predictor of hypertension, especially in populations with different BMI, needs further studies.

## AUTHOR CONTRIBUTIONS

**Li Ming**: Protocol development (lead); data acquisition (lead); data curation (equal); data analysis (equal); methodology (lead); writing—original draft (equal); writing—review & editing (lead). **Duan Wang**: Protocol development (lead); data acquisition (equal); data curation (equal); data analysis (equal); methodology (lead); writing—original draft (lead); Writing—review & editing (lead). **Yong Zhu**: Protocol development (lead); data acquisition (lead); data curation (lead); data analysis (equal); methodology (lead); Writing—original draft (lead); Writing—review & editing (lead).

## CONFLICT OF INTEREST STATEMENT

The authors declare no conflict of interest.

## Data Availability

The data sets generated and analyzed during the current study are available in the nhanes repository, (https://wwwn.cdc.gov/nchs/nhanes/Default.aspx).
